# The association between COVID-19-related fear and reported self-harm in a national survey of people with a lifetime history of self-harm

**DOI:** 10.1186/s12888-021-03625-0

**Published:** 2022-02-02

**Authors:** Chris Keyworth, Leah Quinlivan, Jess Z. Leather, Rory C. O’Connor, Christopher J. Armitage

**Affiliations:** 1 School of Psychology, University of Leeds, Leeds, UK; 2 NIHR Greater Manchester Patient Safety Translational Research Centre, University of Manchester, Manchester, UK; 3 Manchester Centre for Health Psychology, University of Manchester, Manchester, UK; 4 Suicidal Behaviour Research Laboratory, Institute of Health and Wellbeing, University of Glasgow, Glasgow, UK; 5 Manchester Academic Health Science Centre, Manchester University Foundation Trust, Manchester, M13 9PL UK

## Abstract

**Background:**

Relatively little is known about the characteristics of people living in the community who have previously self-harmed and may benefit from interventions during and after COVID-19. We therefore aimed to: (a) examine the relationship between reported self-harm and COVID-19-related fear, and (b) describe the characteristics of a community sample of people who reported a lifetime history of self-harm.

**Methods:**

A cross-sectional national online survey of UK adults who reported a lifetime history of self-harm (*n* = 1029) was conducted. Data were collected May – June 2020. Main outcomes were self-reported COVID-19-related fear (based on the Fear of COVID-19 scale [FCV-19S]), lifetime history of COVID-19, and lifetime history of self-harm. Data were analysed using descriptive statistics and binary logistic regression. Chi-square was used to compare characteristics of our sample with available national data.

**Results:**

Overall, 75.1, 40.2 and 74.3% of the total sample reported lifetime suicidal ideation, suicidal attempts and non-suicidal self-harm respectively. When adjusting for age, sex, ethnicity, social grade, and exposure to death and suicide, binary logistic regression showed higher levels of perceived symptomatic (or physiological) reactions to COVID-19 were associated with suicidal ideation (OR = 1.22, 95%CI 1.07, 1.39) and suicidal attempts (OR = 3.91, 95%CI 1.18, 12.96) in the past week.

**Conclusions:**

Results suggest an urgent need to consider the impact of COVID-19 on people with a lifetime history of self-harm when designing interventions to help support people in reducing suicidal ideation and suicidal attempts. Experiencing symptomatic reactions of fear in particular is associated with self-harm. Helping to support people to develop coping plans in response to threat-related fear is likely to help people at risk of repeat self-harm during public health emergencies.

## Background

The COVID-19 pandemic has major impacts for population mental health [1, 2]. However little is known about the impacts of the COVID-19 pandemic on people with a lifetime history of self-harm, who may be particularly badly affected by COVID-19 and its associated containment measures such as self-isolation and physical distancing. The potential mental health and psychological consequences of COVID-19 containment measures are well documented [3], including the potential impacts on suicide and self-harm [4]. There are also growing concerns that the COVID-19 pandemic, and its related containment measures may also lead to additional self-harm [5] over and above established risk factors including age [6], gender [7], ethnicity [8], and social background [9]. In particular, many of the COVID-19-related challenges, including high prevalence of self-reported mental health challenges, physical health challenges, economic uncertainty and job insecurity [2], extended periods of loneliness and isolation [10], and disruption to mental health services [11], are associated with higher rates of self-harm and suicide [12].

An additional concern is that reductions in attendance at primary care settings for people who harmed themselves during the initial COVID-19 pandemic restrictions in the UK, could lead to further presentations of self-harm and suicide [13]. Self-harm may include: self-harm with suicidal intent (suicidal attempts), self-harm without suicidal intent (non-suicidal self-harm) or suicidal thoughts/ideation [14]. The main aim of the present study is to estimate the impacts of COVID-19 on people who have previously self-harmed, a group that is commonly compared with the general population [13, 15, 16] but never examined in sufficient numbers in its own right.

The potentially detrimental impacts of COVID-19 on people who have previously self-harmed may be wide-ranging; however, there are three areas of uncertainty. First, little is known about the impacts of the COVID-19 pandemic on rates of self-harm [17]. Early findings from a living systematic review shows that due to a lack of high quality studies, there is currently no clear evidence of an increase in rates of self-harm associated with the onset of the COVID-19 pandemic, nor with the associated containment measures [16].

Second, no studies to-date have examined the impact of recent history of self-harm on reported fear of COVID-19. A limitation of the studies in John et al’s review is the use of generic measures of fear, anxiety and depression (often using a single item), and a lack of COVID-19-specific measurements [16]. This is important because identifying COVID-19-specific concerns will lead to greater precision in future intervention development.

Third, few studies have characterised community samples of UK adults with a lifetime history of self-harm in any depth. This is important because knowing more about community populations with a lifetime history of self-harm allows more targeted preventative strategies, with respect to specific sub-groups who may benefit the most from interventions. Previous general population studies are limited as they do not provide a sufficient understanding of the characteristics of people who have previously self-harmed, nor do they examine self-harm across the lifespan, with previous research focusing on younger adults [14]. The small numbers of people reporting self-harm in previous studies [13, 15] means limited conclusions can be made about the characteristics of people reporting self-harm; McManus et al. report lifetime prevalence of non-suicidal self-harm of 2.4, 3.8, and 6.4% in three surveys conducted in 2000, 2007, and 2014 respectively [15].

Consequently, little is currently known about the characteristics of community samples with a lifetime history of self-harm. Whilst McManus et al. measure suicidal ideation, suicidal attempts, and non-suicidal self-harm [18], the measures used do not: (a) take into account frequency or recency of self-harm (only in the most recent 2014 survey was there a measure of recency of non-suicidal self-harm), or (b) measure exposure to suicidal behaviour of others (family or friends) which is recognised as a risk factor for suicidal behaviour [19]. To address the gaps in the literature, this study aimed to characterise a national community sample of adults who have previously self-harmed with respect to: demographic variables, history of non-suicidal self-harm, suicidal ideation and suicidal attempts, and exposure to suicide and death. This is necessary to ensure that interventions can be targeted at the people who are most likely to benefit from them.

Given the sharp decrease in presentations for self-harm in primary care settings following the onset of the COVID-19 pandemic, compared to expected rates [13], it would be valuable to gauge potential harms in this population. There is still uncertainty surrounding an increase in self-harm referrals in the aftermath of COVID-19 [20]. However, identifying the relationship between recent history of self-harm and reported fear of COVID-19, would allow us to determine whether people with a recent history of self-harm are more or less resilient to COVID-19-related stressors, or whether COVID-19 has detrimental impacts on an already vulnerable group. Based on the gaps identified in the literature, this study aimed to: (a) examine the relationship between self-harm outcomes and COVID-19-related fear, and (b) provide in-depth characteristics of a national community sample of adults who have previously self-harmed.

## Methods

### Design and procedure

The study was part of a wider survey testing the acceptability of a psychological intervention to reduce self-harm [21] (ClinicalTrials.gov Identifier: NCT04420546). The analyses use baseline data collected in June 2020, approximately 1 month after the first full lockdown in the UK was eased, including the phased re-opening of schools (from 1 June), and the re-opening of non-essential shops (from 15 June) [22]. A sample of adults with a lifetime history of self-harm was invited to take part in an online questionnaire distributed by YouGov, an online survey panel company. Participants (who were current members of YouGovs panel) were incentivised in accordance with YouGov’s points system, whereby respondents accumulate points for taking part in online surveys. Data were sent securely to the research team for analysis. Ethical approval was obtained from a University Research Ethics Committee (ref: 2020–8446-15,312) and participants gave informed consent at the beginning of the survey. Initially, a sample designed to be representative of adults resident in the United Kingdom was asked a screening question to ensure the sample contained people with a lifetime history of self-harm, a screening question was asked: “have you ever intentionally hurt yourself/ self-harmed?”. Response options were: “yes, I have”, “no, I haven’t”, or “prefer not to say”. The final sample was based on respondents answering “yes, I have”.

### Measures

#### Sociodemographic variables

Demographic variables included age, sex, ethnicity, and social grade were taken using standard UK Office for National Statistics [23] measures.

#### History of non-suicidal self-harm (NSSH), suicidal ideation and suicide attempts

Three items drawn from the British Psychiatric Morbidity Survey [24]: “Have you ever seriously thought of taking your life, but not actually attempted to do so?” (suicidal ideation), “Have you ever made an attempt to take your life, by taking an overdose of tablets or in some other way?” (suicidal attempt), and “Have you ever deliberately harmed yourself in any way but not with the intention of killing yourself? (i.e., self-harm)” (NSSH). Response options for all questions were “Yes”, “No”, or “prefer not to say”. If respondents answer yes to any of the three questions, participants were asked when the last episode occurred and with what frequency (past week/past year).

#### Exposure to death and suicidal behaviour

Participants were asked to complete seven items [19, 25] to establish whether any of their close friends or family had died, whether they had friends or family who had self-harmed, or who attempted or died by suicide (e.g. “Has anyone among your family attempted suicide?”).

#### COVID-19-related measures

Participants completed The Fear of Coronavirus-19 Scale [26], which assesses participants’ agreement with seven items (e.g., “I cannot sleep because I am worried about getting coronavirus-19”) with respect to fear of COVID-19. Participants were asked to respond on a 5-point scale (*strongly* disagree [1]-*strongly agree* [10]). A total score (as a continuous variable) was calculated by adding each item together (range 7–35), with higher scores corresponding to higher perceived fear of COVID-19. Previous research has also suggested a two-factor model of the Fear of Coronavirus-19 Scale [26–28], with two distinct corresponding sub-scales, namely, emotional fear reactions (e.g. “It makes me uncomfortable to think about the coronavirus”) and symptomatic (or physiological) expressions of fear (e.g. “My hands become clammy when I think about the coronavirus”). Tzur Bitan et al. found that a two-factor model explains a large proportion of the total variance observed in reported COVID-19-related fear (53.71 and 12.05% respectively) [27]. Therefore, scores were also calculated for the two corresponding subscales. Participants were also asked to self-report their lifetime history of COVID-19 with the item “What is your current COVID-19 status?” (e.g. “Definitely think I had COVID-19 but not confirmed with a test”; response options are provided in Table 1).

**Table 1:** Sample demographics (*n* = 1029)

Variable	*n*	*%*	*Mean*	*SD*	*Range*	*General population data*^*a*^	*χ* ^*2*^ *for difference between sample and population*
Sex							
Women	671	65.2				54.5	3.14 (*p* = .08)
Men	340	33.0				45.5	3.14 (*p* = .08)
Other/prefer not to say	18	1.8					
Age^b^			45.55	14.23	18–87		
18–24	57	5.5				10.3	1.09 (*p* = .30)
25–34	228	22.2				16.1	1.17 (*p* = .28)
35–44	227	22.1				17.8	0.50 (*p* = .48)
45–54	196	19.0				21.1	0.13 (*p* = .72)
55–64	217	21.1				19.1	0.13 (*p* = .72)
65–74	93	9.0				9.6	0.00 (*p* = 1.00)
75>	11	1.1				5.9	3.70 (*p* = .10)
Ethnicity							
White	931	90.5				87.1	0.82 (*p* = .37)
Black, Asian and minority ethnic	36	3.5				12.9	5.21 (*p* < .05)
Prefer not to say	62	6.0					
Social grade							
Non-manual worker	645	62.7				–	–
Manual / unemployed	384	37.3				–	–
Suicidal ideation (Ever)	773	75.1				20.6	58.41 (*p* < .001)
Past week	80	10.3				–	–
Past year	247	32.0				5.4	24.18 (*p* < .001)
Longer ago	438	56.7				–	–
Would rather not say / Did not answer	8	1.0				–	–
Suicidal attempt (Ever)	414	40.2				6.7	30.29 (*p* < .001)
Past week	4	1.0				–	–
Past year	39	9.4				0.7	6.74 (*p* < .05)
Longer ago	366	88.4				–	–
Would rather not say / Did not answer	5	1.2				–	–
Non-suicidal self-harm (Ever)	765	74.3				7.3	93.14 (*p* < .001)
Past week	55	7.2				–	–
Past year	150	19.6				–	–
Longer ago	551	72.1				–	–
Would rather not say / Did not answer	8	1.0				–	–
Exposure to suicide and death							
Exposure to death (immediate family)	529	51.4				–	–
Exposure to death (close friend or relative)	779	75.7				–	–
Exposure to death by suicide (family or close friend)	304	29.5				–	–
Suicidal attempt (in the family)	369	35.9				–	–
Suicidal attempt (by close friends)	376	36.5				–	–
NSSH (in the family)	344	33.4				–	–
NSSH (by close friends)	437	42.5				–	–
Lifetime history of COVID-19 (self-reported)							
Definitely not had COVID-19 and had it confirmed with a test	59	5.7				–	–
Definitely think I didn’t have COVID-19 but not confirmed with a test	649	63.1				–	–
Might have had COVID-19	227	22.1				–	–
Definitely think I had COVID-19 but not confirmed with a test	86	8.4				–	–
Definitely had COVID-19 and had it confirmed with a test	8	0.8				–	–
Fear of COVID-19 scale			17.20	6.38	7–35	–	–
Fear of COVID-19 (emotional reaction sub-scale)			11.51	4.18	4–20	–	–
Fear of COVID-19 (symptomatic reaction sub-scale)			5.69	2.70	3–15	–	–

^a^Data retrieved from the Adult Psychiatric Morbidity Survey (APMS) 2014 (Prevalence and recency of lifetime suicidal thoughts, suicide attempts and self-harm). Prevalence rates according to people who report lifetime history of self-harm (ever) on any measure (NSSH, suicidal thoughts, or suicidal attempts). Prevalence rates for self-harm outcomes relates to general population prevalence rates

^b^Categories according to the Adult Psychiatric Morbidity Survey (APMS) 2014

#### Analyses

Descriptive statistics were used to summarise sociodemographic variables, the prevalence and characteristics of suicidal ideation, suicidal attempts, NSSH, exposure to suicidal behaviour and death, lifetime history of COVID-19, and self-reported fear of COVID-19. Chi-square was used to compare our sample of people who reported a lifetime history of self-harm with general population data collected as part of the Adult Psychiatric Morbidity Survey [15]. Binary logistic regression analyses were used to examine associations between COVID-19-related factors (lifetime history of COVID-19 and Fear of COVID-19 [emotional fear reactions and symptomatic expressions of fear [27]]), and self-harm outcomes (suicidal ideation in the past week, Suicidal attempt in the past week, and NSSH in the past week). We adjusted for potentially confounding factors and known predictors of self-harm: age, sex, ethnicity, social grade, and exposure to death and suicidal behaviour (friends and family). The variables sex, ethnicity, social grade, and exposure to death and suicide were coded as binary variables, and age was a continuous variable. With respect to COVID-19-related variables, lifetime history of COVID-19 was coded as a binary independent variable, and Fear of COVID-19 (and the two corresponding sub-scales [emotional reactions and symptomatic reactions]) were coded as continuous variables. All self-harm outcomes were coded as binary outcomes (e.g. *self-harm in the past week* [1] or *no self-harm in the past week*[0]). This timeframe was used to allow us to examine the impact of COVID-19 on self-harm outcomes.

## Results

### Sample characteristics

The total sample (*n* = 1029) comprised mostly women (65.2%), and a mean age of 45.55 years (*SD* = 14.23). The majority of the sample was White (90.5%), and 62.7% were of higher social grade (non-manual worker). Table 1 shows an overview of our sample compared to national data (where available). Characteristics of our sample closely resembled the characteristics of people who reported a lifetime of self-harm according to the Adult Psychiatric Morbidity Survey of the general population [15] in terms of sex and age. However, our sample contained a lower proportion of people from Black, Asian, and minority ethnic backgrounds, compared to national data.

### Prevalence of suicidal ideation, suicidal attempts, non-suicidal self-harm, and exposure to suicidal behaviour and death

Overall, 75.1, 40.2 and 74.3% of the total sample reported suicidal ideation, suicidal attempts and NSSH respectively (Table 1). Further, 10.3% of the total sample reported suicidal thoughts in the past week, and 32% of the sample reported suicidal thoughts in the past year. Few people reported suicidal attempts in the past week (1.0%), and 9.4% reported a suicidal attempt in the past year. With respect to NSSH, 7.2% reported NSSH in the past week, and 19.6% reported NSSH in the past year.

Over half the sample (51.4%) reported experiencing the death of a family member, over three quarters of the sample reported experience of the death of a close friend or relative, and 29.5% of the sample reported experience of death by suicide of a close friend or relative. Of the total sample, 35.9% reported exposure to a family member making a suicidal attempt, and 36.5% reported exposure to a suicidal attempt by a close friend. Exposure to NSSH by a family member was reported by 33.4% of the sample, and NSSH by a close friend by 42.5% of the sample.

With respect to lifetime history of self-harm, our sample reported higher prevalence of suicidal ideation (75.1% versus 20.6%), suicidal attempts (40.2% versus 6.7%), and non-suicidal self-harm (74.3% versus 7.3%) compared to national data. With respect to self-harm in the previous year, our sample reported higher prevalence of suicidal ideation (32.0% versus 5.4%) and suicidal attempts (9.4% versus 0.7%) compared to national data.

Self-reported “Fear of COVID-19” was relatively modest, with scores averaging 17.20 (*SD* = 6.38), out of a maximum score of 35. Similar findings were observed for the two sub-scales: emotional reactions (*M* = 11.51, *SD* = 4.18, out of a maximum score of 20) and symptomatic reactions (*M* = 5.69, *SD* = 2.70, out of a maximum score of 15). However, 30.4% (*n* = 313) of our sample reported that they might have had COVID-19, which was substantially higher than most estimates of infection rates, the larger of which estimated around an 18.1% infection rate as of 7th May 2020 [29].

### Associations between COVID-19-related factors and suicidal and self-harm outcomes

Table 2 shows the binary logistic regression results of associations between COVID-19-related factors (lifetime history of COVID-19 and Fear of COVID-19 [emotional fear reactions and symptomatic expressions of fear]), and self-harm outcomes. Suicidal ideation in the past week was associated with lower levels of perceived emotional fear reactions to COVID-19 (OR = 0.91, 95%CI 0.84–0.99). Higher levels of perceived symptomatic reactions to COVID-19 were associated with suicidal ideation (OR = 1.22, 95%CI 1.07, 1.39) and suicidal attempts (OR = 3.91, 95%CI 1.18, 12.96) in the past week but not non-suicidal self-harm.

**Table 2:** Logistic regression analysis for predictors of self-harm in the past week (adjusted for age, sex, ethnicity, social grade, and exposure to death or suicide)

	Self-harm past week Odds Ratio (95%CI)		
	Suicidal ideation	Suicidal attempt	NSSH
	OR (95%CI)	OR (95%CI)	OR (95%CI)
Lifetime history of COVID-19 (yes)^a^	0.96 (0.57, 1.62)	2.67 (0.13, 53.59)	0.67 (0.35, 1.27)
Fear of COVID-19 (emotional reaction sub-scale)	0.91* (0.84, 0.99)	0.39 (0.14, 1.05)	0.95 (0.86, 1.05)
Fear of COVID-19 (symptomatic reaction sub-scale)	1.22** (1.07, 1.39)	3.91* (1.18, 12.96)	1.15 (0.99, 1.33)

Note: age, sex, ethnicity, social grade, and exposure to death or suicide were all non-significant in the final regression models

*OR* odds ratio, *95%CI* 95% confidence interval

^a^Dichotomised according to: “definitely not had COVID-19 and had it confirmed with a test”, “definitely think I didn’t have COVID-19 but not confirmed with a test” (no), and “might have had COVID-19”, “definitely think I had COVID-19 but not confirmed with a test”, “definitely had COVID-19 and had it confirmed with a test” (yes)

*p < .05 **p < .01

## Discussion

This study aimed to examine the impacts of COVID-19-related fear and lifetime history of COVID-19 on people who have previously self-harmed. This is the first study to: (a) deploy COVID-19-specific measures to examine the impact of COVID-19 on self-harm outcomes, and (b) provide in-depth characteristics of a national community sample of adults who have previously self-harmed with respect to: demographic variables, history of non-suicidal self-harm, suicidal ideation and suicidal attempts, and exposure to death and suicide. There are two important findings. First, COVID-19-specific fear is associated with self-harm outcomes. People experiencing greater COVID-19-specific emotional expressions of fear were less likely to report suicidal ideation; conversely, people experiencing greater COVID-19-specific symptomatic expressions of fear were more likely to report suicidal ideation and a suicidal attempt in the past week (although our results with respect to suicidal attempts in the past week should be noted with caution given the very low number of respondents reporting a suicidal attempt in the past week [*n* = 4]). Second, rates of suicidal ideation, suicidal attempts and non-suicidal self-harm were higher than in the national Adult Psychiatric Morbidity Survey (20.6% versus 75.1, 6.7% versus 40.2, and 7.3% versus 74.3% respectively) [18]. Whilst the higher rates observed in our study may be a consequence of COVID-19 containment measures (social and physical distancing measures are themselves risk factors for suicide and self-harm [30, 31]), nevertheless, our findings suggest rates of self-harm in the community may be higher than some national surveys suggest. Therefore, interventions aimed at reducing self-harm should be prioritised, as well as those aiming to address COVID-19-related fear.

### Implications

Findings demonstrate the need to target COVID-19-specific fears as part of treatment programmes for people with a lifetime history of self-harm. Our findings show that COVID-19-specific fear is associated with self-harm outcomes whilst controlling for known risk factors including age, sex, ethnicity, social grade, and exposure to death or suicide. Future research should aim to build on these findings in order to determine whether reducing COVID-19-specific fear is associated with a reduction in suicidal ideation and suicidal attempts. Further, it would be valuable to examine the role of COVID-19-specific stressors on rates of self-harm and suicide, including economic uncertainty and job insecurity [2], extended periods of loneliness and isolation [10], and disruption to mental health services [11], which are associated with self-harm and suicide [12]. Knowing more about this community population would also allow more targeted preventative strategies for self-harm. One approach might be to incorporate specific behaviour change interventions into treatment programmes that can be used as part of patient healthcare [32, 33], in order to help support people to develop effective coping plans when experiencing COVID-19-specific fear. Emotional regulation strategies such as reappraising the situation surrounding a pandemic have yielded promising effects on producing less fear and consequently better long-term mental health outcomes [34–36]. However, such strategies must be considered with caution given the mixed findings to-date, with respect to the effects of reappraisal- based interventions on health behaviours and compliance with COVID-19 containment measures [35, 36].

### Strengths and limitations

A strength of the present study was the use of COVID-19-specific measurements to examine levels of fear in people who report a lifetime history of self-harm, as opposed to more general measures of fear and anxiety reported used in recent studies [16]. This is important because using COVID-19-specific measures enables researchers to develop more precisely interventions to mitigate the specific impact of COVID-19 on rates of self-harm. Our findings suggest that whilst symptomatic fear reactions to COVID-19 may increase the likelihood of self-harm, some level of fear (i.e. emotional reactions) appears to be a protective factor for suicidal ideation. This is in line with the wider health communication literature showing that some level of fear can motivate protective behaviours [37, 38].

There are limitations to this study. Participants were identified through a pre-existing sample of the general public who were recruited and incentivised by YouGov to take part in the research. Whilst participants were screened in order to ensure all respondents had a lifetime history of self-harming, the sample therefore may not be fully representative of all people who have recently self-harmed. However, YouGov attempted to overcome this by seeking the widest possible variation in terms of demographic characteristics, according to people who reported a lifetime history of self-harm.

Due to a lack of available studies among community samples with a lifetime history of self-harm, we were unable to determine whether our sample is representative of this population. However, we were able to compare our sample with data from the Adult Psychiatric Morbidity Survey of the general population to compare demographic characteristics and self-harm outcomes among people who report a lifetime history of self-harm. Our sample closely resembled the Adult Psychiatric Morbidity Survey data [15] in terms of sex and age. However, our sample contained a lower proportion of people from a minority ethnic background, compared to national data. Our sample also reported higher prevalence of suicidal ideation (lifetime and past year), suicidal attempts (lifetime and past year), and non-suicidal self-harm (lifetime) compared to national data. We were unable to identify data on self-harm outcomes in the past week and non-suicidal self-harm outcomes in the past week or past year. The cross-sectional nature of the study meant that we were unable to assess: (a) the onset of self-harm outcomes, or (b) any changes in COVID-19-related fear. This is particularly important given reported fear experienced during different stages of a pandemic is likely to change as government measures are relaxed, and later reintroduced. Future studies would therefore benefit from examining changes in COVID-19-related fear over time.

## Conclusions

The present study suggests an urgent need to consider the impact of COVID-19 on people with a lifetime history of self-harm, as part of interventions to help support people in reducing self-harm. This may include the design of brief interventions for self-harm, and investment in support services for self-harm, particularly those that can be delivered remotely during the pandemic. Our findings suggest that experiencing symptomatic fear reactions in particular is associated with self-harm. Helping to support people to develop coping plans in response to COVID-19-related fear is likely to help people reduce the likelihood of repeat self-harm among vulnerable populations during a health emergency.

## Data Availability

The datasets used and/or analyzed during the current study are available from the corresponding author on reasonable request.

## References

[1] Gunnell D, Appleby L, Arensman E, Hawton K, John A, Kapur N (2020). Suicide risk and prevention during the COVID-19 pandemic. Lancet Psychiatry.

[2] Keyworth C, Epton T, Byrne-Davis L, Leather JZ, Armitage CJ (2021). What challenges do UK adults face when adhering to COVID-19-related instructions? Cross-sectional survey in a representative sample. Prev Med.

[3] Brooks SK, Webster RK, Smith LE, Woodland L, Wessely S, Greenberg N (2020). The psychological impact of quarantine and how to reduce it: rapid review of the evidence. Lancet.

[4] Holmes EA, O'Connor RC, Perry VH, Tracey I, Wessely S, Arseneault L (2020). Multidisciplinary research priorities for the COVID-19 pandemic: a call for action for mental health science. Lancet Psychiatry.

[5] Xin M, Luo S, She R, Yu Y, Li L, Wang S (2020). Negative cognitive and psychological correlates of mandatory quarantine during the initial COVID-19 outbreak in China. Am Psychol.

[6] Carr MJ, Ashcroft DM, Kontopantelis E, Awenat Y, Cooper J, Chew-Graham C (2016). The epidemiology of self-harm in a UK-wide primary care patient cohort, 2001–2013. BMC Psychiatry.

[7] Nock MK, Borges G, Bromet EJ, Alonso J, Angermeyer M, Beautrais A (2008). Cross-national prevalence and risk factors for suicidal ideation, plans and attempts. Br J Psychiatry.

[8] Hawton K, Bergen H, Casey D, Simkin S, Palmer B, Cooper J (2007). Self-harm in England: a tale of three cities. Soc Psychiatry Psychiatr Epidemiol.

[9] O'Connor RC, Wetherall K, Cleare S, McClelland H, Melson AJ, Niedzwiedz CL, et al. Mental health and well-being during the COVID-19 pandemic: longitudinal analyses of adults in the UK COVID-19 mental health and wellbeing study. Br J Psychiatry. 2020;218(6):1–8. 10.1192/bjp.2020.212 PMC7684009 33081860

[10] Robb CE, de Jager CA, Ahmadi-Abhari S, Giannakopoulou P, Udeh-Momoh C, McKeand J (2020). Associations of Social Isolation with Anxiety and Depression During the Early COVID-19 Pandemic: A Survey of Older Adults in London, UK. Front Psychiatry.

[11] Usher K, Bhullar N, Durkin J, Gyamfi N, Jackson D (2020). Family violence and COVID-19: Increased vulnerability and reduced options for support. Int J Ment Health Nurs.

[12] McManus S, Lubian K, Bennett C, Turley C, Porter L, Gill V (2019). Suicide and self-harm in Britain: researching risk and resilience using UK surveys.

[13] Carr MJ, Steeg S, Webb RT, Kapur N, Chew-Graham CA, Abel KM (2021). Effects of the COVID-19 pandemic on primary care-recorded mental illness and self-harm episodes in the UK: a population-based cohort study. Lancet Public Health.

[14] O'Connor RC, Wetherall K, Cleare S, Eschle S, Drummond J, Ferguson E, et al. Suicide attempts and non-suicidal self-harm: national prevalence study of young adults. BJPsych Open. 2018; 4(3):142–148 . Available from: http://europepmc.org/abstract/MED/29922479, 10.1192/bjo.2018.14, https://europepmc.org/articles/PMC6003254, https://europepmc.org/articles/PMC6003254?pdf=render. 10.1192/bjo.2018.14 PMC6003254 29922479

[15] McManus S, Gunnell D, Cooper C, Bebbington PE, Howard LM, Brugha T (2019). Prevalence of non-suicidal self-harm and service contact in England, 2000-14: repeated cross-sectional surveys of the general population. Lancet Psychiatry.

[16] John A, Okolie C, Eyles E, Webb R, Schmidt L, McGuiness L, et al. The impact of the COVID-19 pandemic on self-harm and suicidal behaviour: a living systematic review [version 1; peer review: 1 approved, 2 approved with reservations]. F1000Research. 2020;9(1097). 10.12688/f1000research.25522.1 PMC7871358 33604025

[17] Kapur N, Clements C, Appleby L, Hawton K, Steeg S, Waters K (2021). Effects of the COVID-19 pandemic on self-harm. Lancet Psychiatry.

[18] McManus S, Bebbington PE, Jenkins R, Brugha T (2016). Mental health and wellbeing in England: the adult psychiatric morbidity survey 2014: NHS digital.

[19] Dhingra K, Boduszek D, O’Connor RC (2015). Differentiating suicide attempters from suicide ideators using the integrated motivational–volitional model of suicidal behaviour. J Affect Disord.

[20] John A, Pirkis J, Gunnell D, Appleby L, Morrissey J (2020). Trends in suicide during the covid-19 pandemic. BMJ..

[21] Keyworth C, O'Connor R, Quinlivan L, Armitage CJ. Acceptability of a brief web-based theory-based intervention to prevent and reduce self-harm: mixed methods evaluation. J Med Internet Res. 2021;23(9):e28349. 10.2196/28349 PMC8479604 34518153

[22] lockdowns IfGJToUc (2021). Timeline of UK Coronavirus lockdowns, March 2020 to March 2021.

[23] Statistics UOfN. 2020 [Available from: https://www.ons.gov.uk/peoplepopulationandcommunity/birthsdeathsandmarriages/deaths/bulletins/deathsinvolvingcovid19bylocalareasanddeprivation/deathsoccurringbetween1marchand31july2020.

[24] Nicholson S, Jenkins R, Meltzer H (2009). Adult psychiatric morbidity in England, 2007.

[25] O'Connor RC, Rasmussen S, Hawton K (2012). Distinguishing adolescents who think about self-harm from those who engage in self-harm. Br J Psychiatry.

[26] Ahorsu DK, Lin C-Y, Imani V, Saffari M, Griffiths MD, Pakpour AH. The fear of COVID-19 scale: development and initial validation. Int J Ment Health Addict. 2020:1–9. 10.1007/s11469-020-00270-8 PMC7100496 32226353

[27] Tzur Bitan D, Grossman-Giron A, Bloch Y, Mayer Y, Shiffman N, Mendlovic S (2020). Fear of COVID-19 scale: Psychometric characteristics, reliability and validity in the Israeli population. Psychiatry Res.

[28] Reznik A, Gritsenko V, Konstantinov V, Khamenka N, Isralowitz R. COVID-19 fear in Eastern Europe: validation of the fear of COVID-19 scale. Int J Ment Health Addict. 2020;1–6. 10.1007/s11469-020-00283-3 PMC7217343 32406404

[29] Sturgis P, Kuha J (2020). Estimating how many Britons have already had COVID-19 using self-reported data.

[30] O'Connor RC, Nock MK (2014). The psychology of suicidal behaviour. Lancet Psychiatry.

[31] John A, Glendenning AC, Marchant A, Montgomery P, Stewart A, Wood S (2018). Self-harm, suicidal Behaviours, and Cyberbullying in children and young people: systematic review. J Med Internet Res.

[32] Armitage CJ, Rahim WA, Rowe R, O'Connor RC (2016). An exploratory randomised trial of a simple, brief psychological intervention to reduce subsequent suicidal ideation and behaviour in patients admitted to hospital for self-harm. Br J Psychiatry.

[33] O'Connor RC, Ferguson E, Scott F, Smyth R, McDaid D, Park AL (2017). A brief psychological intervention to reduce repetition of self-harm in patients admitted to hospital following a suicide attempt: a randomised controlled trial. Lancet Psychiatry.

[34] Low RS, Overall N, Chang V, Henderson AM, Sibley CG (2020). Emotion regulation and psychological and physical health during a nationwide COVID-19 lockdown.

[35] Wang K, Goldenberg A, Dorison CA, Miller JK, Uusberg A, Lerner JS (2021). A multi-country test of brief reappraisal interventions on emotions during the COVID-19 pandemic. Nat Hum Behav.

[36] Smith AM, Willroth EC, Gatchpazian A, Shallcross AJ, Feinberg M, Ford BQ (2021). Coping With Health Threats: The Costs and Benefits of Managing Emotions. Psychol Sci.

[37] Rubin GJ, Potts HW, Michie S (2010). The impact of communications about swine flu (influenza A H1N1v) on public responses to the outbreak: results from 36 national telephone surveys in the UK. Health Technol Asses.

[38] Chater AM, Arden M, Armitage C, Byrne-Davis L, Chadwick P, Drury J, et al. Behavioural science and disease prevention: psychological guidance. Br Psychol Soc. 2020.

